# Neoadjuvant chemotherapy followed by concurrent chemoradiotherapy with or without nimotuzumab in the treatment of locally advanced nasopharyngeal carcinoma: a retrospective study

**DOI:** 10.1186/s12885-023-11608-5

**Published:** 2023-11-24

**Authors:** Zhi Yang, Quan Zuo, Rong Liu, Hui Wu, Li Xiong, Jieqi Jia, Zhibi Xiang

**Affiliations:** 1 Department of Oncology, People’s Hospital of Xiangxi Tujia and Miao Autonomous Prefecture, First Affiliated Hospital of Jishou University, Jishou, 416000 Hunan China; 2 Department of Otolaryngology, People’s Hospital of Xiangxi Tujia and Miao Autonomous Prefecture, First Affiliated Hospital of Jishou University, Jishou, 416000 Hunan China

**Keywords:** Nasopharyngeal carcinoma, Neoadjuvant chemotherapy, Concurrent chemoradiotherapy, Nimotuzumab

## Abstract

**Purpose:**

We aimed to investigate the efficacy and side effects of concurrent chemoradiotherapy, with or without nimotuzumab, for the treatment of locally advanced nasopharyngeal carcinoma after neoadjuvant chemotherapy.

**Methods:**

This study retrospectively enrolled 109 patients with NPC from our hospital from July 2019 to May 2021.All patients were treated with docetaxel, cisplatin, and fluorouracil(TPF) neoadjuvant chemotherapy for 2 cycles, and concurrent chemoradiotherapy was performed 2 weeks after chemotherapy. According to whether nimotuzumab was added in concurrent chemoradiotherapy, they were divided into the nimotuzumab group and the control group, with 52 cases in the nimotuzumab group and 57 cases in the control group.The efficacy and adverse reactions of the two groups were retrospectively analyzed.

**Results:**

The objective remission and complete remission rates in the nimotuzumab and control groups were 100% vs 98.2% (*p* = 1.000), and 92.3% vs 78.9% (*p* = 0.049), respectively. The 3-year distant metastasis-free survival of the nimotuzumab and control groups was 91.6% and 77.3% (*p* = 0.047), respectively.The 3-year progression-free survival, locoregional relapse-free survival, and overall survival of the nimotuzumab and control groups were 87.6% vs 75.5% (*p* = 0.110), 90.5% vs 86.9% (*p* = 0.566), and 94.5% vs 87.1% (*p* = 0.295), respectively. In the nimotuzumab group, subgroup analysis showed that patients aged < 60 years (hazard ratio [HR] = 0.350, 95% confidence interval [CI]: 0.131–0.934, *p* = 0.036) and those with a neutrophil-to-lymphocyte ratio (neutrophil/lymphocyte ratio) ≤ 4 (HR = 0.365, 95% CI: 0.144–0.923, *p* = 0.033) achieved a better result. Additionally, multivariate analysis demonstrated that neutrophil/lymphocyte ratio was an independent risk factor for disease progression (HR = 7.485, *p* = 0.012) and distant metastasis (HR = 17.540, *p* = 0.009).No grade 4 adverse reactions were observed in either group. Grade 3 oral mucosal reactions, as well as pharyngeal and esophageal reactions were slightly higher in the nimotuzumab group than in the control group, but the difference was not statistically significant. No significant differences were observed in the incidence of adverse reactions such as leukopenia, HB reduction, thrombocytopenia between the two groups (*P* > 0.05).

**Conclusion:**

The concurrent chemoradiotherapy plus nimotuzumab after neoadjuvant chemotherapy for locally advanced nasopharyngeal carcinoma achieved a higher complete remission rate and significantly improved distant metastasis-free survival compared with concurrent chemoradiotherapy alone. Additionally, an increasing trend was observed in progression-free survival, and the incidence of side effects was similar in both groups.

## Introduction

Nasopharyngeal carcinoma (NPC) is a common malignant tumor in the head and neck region, particularly in China. Approximately 70–85% of patients with NPC are in the locally advanced stage at the time of initial diagnosis [1]. For patients with locally advanced NPC, concurrent chemoradiotherapy is the main treatment modality [2], and has been shown to improve the treatment efficacy sequentially after neoadjuvant chemotherapy in several studies [3–5]. Docetaxel, cisplatin, and fluorouracil (TPF) regimen is the first-line neoadjuvant chemotherapy for locally advanced NPC [6]. Neoadjuvant chemotherapy combined with concurrent chemoradiotherapy has become a type I recommended treatment for locally advanced high-risk NPC (NCCN recommendation) [7]. Although neoadjuvant chemotherapy improves overall efficacy, distant metastasis remains the main cause of treatment failure in locally advanced NPC [8].

High expression of the epidermal growth factor receptor (EGFR) is a poor prognostic factor for tumors [9], and has been observed in 80%-90% of patients with NPC [10]. Nimotuzumab is a non-intrinsically stimulating anti-EGFR monoclonal antibody that blocks the binding of EGFR to its ligand and exhibits anti-angiogenic, anti-tumor cell proliferative, and pro-apoptotic effects in EGFR-overexpressing tumors [11]. Wang et al. found that adding nimotuzumab to concurrent chemoradiotherapy for locally advanced NPC could improve the 5-year distant metastasis-free survival (DMFS) and overall survival (OS) [12]. Under the current neoadjuvant chemotherapy sequential concurrent chemoradiotherapy mode, there is a lack of relevant research on whether concurrent chemoradiotherapy combined with nimotuzumab provides further benefits.The purpose of this study was to investigate the efficacy and adverse reactions of concurrent chemoradiotherapy with or without nimotuzumab in locally advanced nasopharyngeal carcinoma after neoadjuvant chemotherapy, and to provide evidence-based medical evidence for the selection of treatment strategies for locally advanced nasopharyngeal carcinoma.

## Data and methods

### General information

This study included 115 patients with NPC admitted to our hospital from July 2019 to April 2021.The inclusion criteria were follows: (1) age 18–70 years; (2) pathological diagnosis of NPC (including non-keratinizing carcinoma [differentiated and undifferentiated], keratinizing squamous cell carcinoma, basal cell squamous cell carcinoma, and adenocarcinoma and excluding neuroendocrine carcinoma) and immunohistochemically suggested EGFR ( +); (3) NPC diagnosed as T1-2N2M0, T3N1-2M0, T1-3N3M0, and T4N0-3M0 based on imaging examination (the eighth version).The exclusion criteria were as follows: (1) patients with other tumors, such as double primary cancer; (2) The survival time of patients with other diseases ( such as coronary heart disease, cerebral infarction and other serious cardiovascular and cerebrovascular diseases) is shortened..

### Treatment methods

All patients received paclitaxel liposome, cisplatin combined with fluorouracil ( TPF) regimen neoadjuvant chemotherapy and radical concurrent chemoradiotherapy. Immunohistochemical positive patients with locally advanced nasopharyngeal carcinoma after doctors and patients and their families to communicate the condition and treatment, according to the wishes of patients and their families whether to use nimotuzumab in concurrent chemoradiotherapy.According to whether nimotuzumab was added in concurrent chemoradiotherapy, they were divided into the nimotuzumab group and the control group, with 52 cases in the nimotuzumab group and 57 cases in the control group.This study was approved by the ethics committee of our hospital.Patients and their families agreed to this study.

Neoadjuvant chemotherapy: Patients received two cycles of TPF neoadjuvant chemotherapy before chemoradiotherapy with the following regimen: (paclitaxel liposome 135 mg/m2 d1, cisplatin 25 mg/m2 d1-3, fluorouracil 600 mg/m2 continuous intravenous infusion on d1-5, repeated every three weeks).

Concurrent chemoradiotherapy: Two weeks after neoadjuvant chemotherapy, patients in both groups were treated with the same intensity modulated radiation therapy (IMRT) using the same technique. The prescribed dose were as follows: 95% PGTVnx(planning gross target volume nasopharynx) 69.96–73.92 Gy/33f, 95% PGTVnd(planning gross target volume lymph node) 69.96 Gy/33f, 95% PTV1 (planning target volume 1)60.06 Gy/33f, and 95% PTV2(planning target volume 2) 50.4 Gy/28f.

Synchronous treatment: During radiotherapy, patients in the nimotuzumab group received concurrent chemotherapy using single-agent cisplatin (80 mg/m2 for three days, administered every 21 days [Q21D], for two 2 cycles) and concurrent targeted therapy using nimotuzumab (200 mg/m2 once a week for six times), while patients in the control group received concurrent chemotherapy using single-agent cisplatin (80 mg/ m^2^, for three days, Q21D for two cycles).

### Efficacy and observation indicators

Short-term efficacy:The maximum diameter of the tumor was measured through imaging examination and evaluated according to the International Response Evaluation Criteria in Solid Tumor (RECIST) 1.1 standard, in which tumor diameters at baseline and after treatment were compared. The efficacy evaluation included complete response (CR), partial response (PR), stable disease (SD), and progressive disease (PD). The objective response rate (ORR) was defined as CR + PR.

Recent toxicity and side effects experienced by patients during concurrent chemoradiotherapy were recorded and evaluated based on the International Common Adverse Reaction Standard (3rd edition) for toxicity and side effects.

Long-term efficacy, including local recurrence, distant metastasis, and patient survival status, were determined at follow-up. The main endpoints was DMFS.The secondary endpoints were progression-free survival (PFS),OS and locoregional relapse-free survival (LRFS).

### Follow-up observation

The patients were re-examined at specific intervals after the completion of treatment as follows:reviewed every three months within 2 years, at 6-month intervals for the next two to five years, and once every year after five years. The follow-up ended on February 28, 2023, with periods ranging from 20 to 45 months and a median of 32.1 months.

### Statistical analysis

All data were analyzed using Statistical Package for the Social Sciences version 20.0. Enumeration data were expressed as % using χ^2^ test, continuity correction χ^2^ test, or Fisher exact probability method. PFS, LRFS, DMFS, and OS were evaluated with log-rank of univariate analysis and multivariate analysis Cox proportional risk model. The incidence of adverse reactions was analyzed using the chi-square test. *P*-values of < 0.05 was considered significant.

## Results

### Comparison of baseline data between the two groups

Differences in age, sex, pathological type, clinical stage, T stage, N stage, Epstein-Barr virus (EBV) DNA level, neutrophil-to-lymphocyte ratio (neutrophil/lymphocyte ratio), lactate dehydrogenase (LDH), HB, and EGFR expression were not statistically significant (all *P* > 0.05) (Table 1).

**Table 1:** Comparison of baseline characteristics between the two groups

Characteristic	Nimotuzumab group	Control group	χ^2^ value	*P* value
Age(years)			0.344	0.557
<60	45(86.5)	47(82.5)		
≥60	7(13.5)	10(17.5)		
Sex			0.894	0.334
Male	38(73.1)	46(80.7)		
Female	14(26.9)	11(19.3)		
Pathological type			0.937	0.333
Non-keratinizing carcinoma	50 (96.2)	51 (89.5)		
Keratocarcinoma	2 (3.8)	6 (10.5)		
Basal cell carcinoma	0(0.0)	0(0.0)		
Adenocarcinoma	0(0.0)	0(0.0)		
Clinical stage			1.645	0.200
III	10(19.2)	6(10.5)		
IVa	42(80.8)	51(89.5)		
T stage			6.154	0.104
T1	1 (1.9)	5 (8.8)		
T2	7 (13.5)	2 (3.5)		
T3	13 (25.0)	13 (22.8)		
T4	31 (59.6)	37 (64.9)		
N stage			1.366	0.505
N0	0(0.0)	0(0.0)		
N1	1 (1.9)	3 (5.3)		
N2	27 (51.9)	25 (43.9)		
N3	24 (46.2)	29 (50.9)		
EB-DNA(Copyies/ml)			0.918	0.338
<100	21 (40.4)	18 (31.6)		
≥100	31 (59.6)	39 (68.4)		
Neutrophil / Lymphocyte ratio			1.440	0.230
>4	5(9.6)	10(17.5)		
≤4	47(90.4)	47(82.5)		
LDH(U/L)			1.044	0.307
>245	4 (7.7)	1 (1.8)		
≤245	48 (92.3)	56 (98.2)		
HB(g/L)			0.235	0.628
<120	6 (11.5)	4 (7.0)		
≥120	46 (88.5)	53 (93.0)		
EGFR expression			0.154	0.926
+	27 (51.9)	29(50.9)		
++	19 (36.5)	20(35.1)		
+++	6 (11.5)	8 (14.0)		

EB-DNA < 100 Copies / ml is negative, ≥ 100 Copies / ml is positive

### Comparison of short-term efficacy between the two groups

In the nimotuzumab group (52 cases), there were 48 and four cases of CR and PR, respectively, but no cases of SD and PD, with an objective remission rate of 100%. In the control group (57 patients), there were 45, 11, and one case(s) of CR, PR, and SD, respectively, but no cases of PD, with an objective remission rate of 98.2%. Compared with the complete remission rate of the two groups, the nimotuzumab group had a higher complete remission rate, the difference was statistically significant (χ^2^=3.876, *P*=0.049). One patient with SD in the control group had tumor-reduced (Table 2).

**Table 2:** Comparison of short-term efficacy between the two groups

Groups	Total Cases	CR	PR	SD	PD	ORR
		Cases(%)	Cases(%)	Cases(%)	Cases(%)	Cases(%)
Nimotuzumab group	52	48(92.3)	4(7.7)	0	0	52(100.0)
Control group	57	45(78.9)	11(19.3)	1(1.8)	0	56(98.2)
χ^2^value		3.876	3.086	0.000	-	0.000
*P* value		0.049	0.079	1.000	-	1.000

### Comparison of long-term efficacy between the two groups

**Fig. 1:**
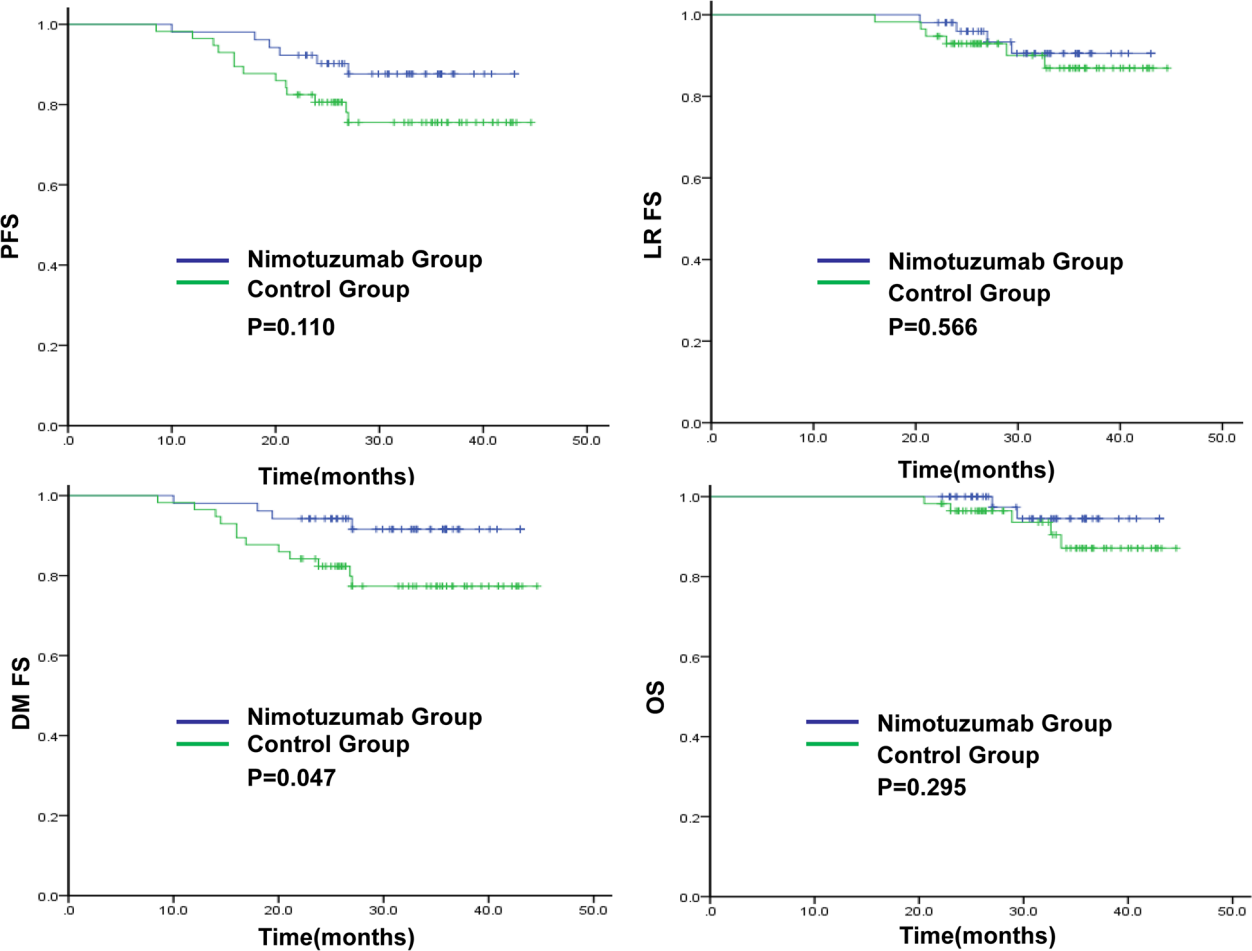
Univariate analysis of PFS, LRFS, DMFS, and OS in the two groups. DMFS, distant metastasis-free survival; LRFS, locoregional relapse-free survival; OS, overall survival; PFS, progression-free survival

The 3-year DMFS rate in the nimotuzumab and control groups was 91.6% and 77.3%(*P* = 0.047), respectively.The 3-year PFS,LRFS and OS rates in the nimotuzumab and control groups were 87.6% vs 75.5% (*P* = 0.110), 90.5% vs 86.9% (*P* = 0.566) and 94.5% vs 87.1% (*P* = 0.295),respectively (Fig. 1). In the nimotuzumab group, there were six cases of disease progression, two of local recurrence, four of distant metastasis, and two of death. In the control group, there were 13 cases of disease progression, two of local recurrence, 12 of distant metastasis, and five cases of death.

**Fig. 2:**
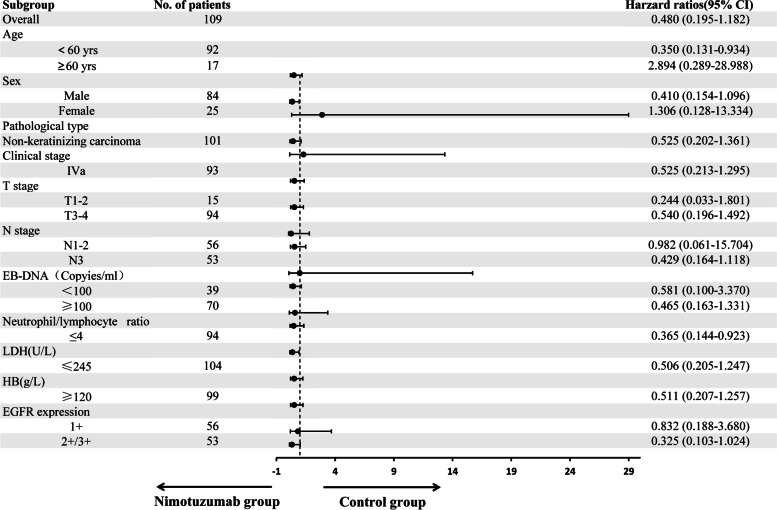
Subgroup analyses of progression-free survival. Groups whose P values or HR values could not be calculated were excluded from the subgroup analysis.CI, confidence interval; EB-DNA, Epstein-Barr virus (EBV) DNA;EGFR, epidermal growth factor receptor; HB, hemoglobin; LDH, lactate dehydrogenase

Subgroup analysis showed in patients with locally advanced NPC after neoadjuvant chemotherapy, concurrent chemoradiotherapy plus nimotuzumab targeted therapy had significant benefits to PFS for individuals aged < 60 years (hazard ratio [HR] = 0.350, 95% confidence interval [CI]: 0.131–0.934, *P*=0.036) and those with neutrophil/lymphocyte ratio ≤ 4 (HR = 0.365, 95% CI: 0.144–0.923, *P* = 0.033). There was a trend towards PFS benefit for patients with male (HR = 0.410, 95% CI: 0.154–1.096, *P* = 0.076),N3 (HR = 0.429, 95% CI: 0.164–1.118, *P* = 0.083), and those with EGFR (+ + / +  +  +  + , HR = 0.325, 95% CI: 0.103–1.024, *P* = 0.055) (Fig. 2).

After radical chemoradiotherapy, EB DNA remained positive in four patients in the control group, while it was negative in the nimotuzumab group. The EB DNA levels after treatment,recent therapeutic effect and baseline factors were considered. Multivariate regression analysis showed that the neutrophil/lymphocyte ratio was an independent risk factor for disease progression (HR = 7.485, *P* = 0.012) and distant metastasis (HR = 17.540, *P* = 0.009) of locally advanced NPC, with a higher neutrophil/lymphocyte ratio being associated with an increased risk of disease progression and distant metastasis after treatment (Table 3).

**Table 3:** Multi-factor regression analysis

Clinical factors	Hazard ratio(95% CI)	*P* value
PFS		
Age	1.209 (0.530–2.758)	0.652
Granulocyte / Lymphocyte ratio	7.485 (1.571–35.661)	0.012
Group ( nimotuzumab group vs control group)	1.448 (0.435–4.820)	0.546
LRFS		
Age	0.140 (0.005–3.938)	0.248
Granulocyte / Lymphocyte ratio	0.469 (0.003–71.627)	0.768
Group ( nimotuzumab group vs control group)	21.307 (0.231–1969.162)	0.185
DMFS		
Age	1.624 (0.629–4.188)	0.316
Granulocyte / Lymphocyte ratio	17.540 (2.019–152.340)	0.009
Group ( nimotuzumab group vs control group)	1.520 (0.425–5.432)	0.520
OS		
Age	4.798 (0.364–63.245)	0.233
Granulocyte / Lymphocyte ratio	693654.379 (0.000–1.729×10^168^)	0.944
Group ( nimotuzumab group vs control group)	15.122 (0.346–661.641)	0.159

### Comparison of adverse reactions between the two groups

No grade 4 adverse reactions were observed in either group. Grade 3 oral mucosal reactions, as well as pharyngeal and esophageal reactions were slightly higher in the nimotuzumab group than in the control group, but the difference was not statistically significant. No significant differences were observed in the incidence of adverse reactions such as leukopenia, HB reduction, thrombocytopenia, elevated alanine aminotransferase (ALT), elevated aspartate aminotransferase (AST), elevated creatinine, nausea, vomiting, radiation dermatitis, weight loss, hyponatremia, hypokalemia, skin rash and infusion reaction between the two groups (*P* > 0.05) (Table 4).

**Table 4:** Comparison of adverse reactions between the two groups

Adverse reactions	Nimotuzumab group(*n*=52)		Control group(*n*=57)		*P* value
	Grade –2(%)	Grade 3–4 (%)	Grade 1–2(%)	Grade 3–4(%)	
Leukopenia	35 (67.3)	1 (1.9)	40 (70.1)	3 (5.3)	0.739
Hemoglobin decreased	20 (38.5)	0 (0.0)	30 (52.6)	0 (0.0)	0.138
Thrombocytopenia	4 (7.7)	0 (0.0)	6 (10.5)	2 (3.5)	0.515
ALT increased	9 (17.3)	0 (0.0)	16 (28.1)	0 (0.0)	0.182
AST increased	2 (3.8)	0 (0.0)	5 (8.8)	0 (0.0)	0.511
Urea increased	2 (3.8)	0 (0.0)	2 (3.5)	0 (0.0)	0.925
Nausea	18 (34.6)	0 (0.0)	17 (29.8)	0 (0.0)	0.593
Vomiting	7 (13.5)	0 (0.0)	11 (19.3)	0 (0.0)	0.412
Oral mucositis	44 (84.6)	8 (15.4)	50 (87.7)	7 (12.3)	0.638
Radiodermatitis	51 (98.1)	1 (1.9)	56 (98.2)	1 (1.8)	1.000
Pharyngeal and esophageal reactions	49 (94.2)	3 (5.8)	56 (98.2)	1 (1.8)	0.546
Weight loss	52 (100.0)	0 (0.0)	57 (100.0)	0 (0.0)	-
Hyponatremia	4 (7.7)	0 (0.0)	3 (5.3)	0 (0.0)	0.900
Hypokalemia	17 (32.7)	2 (3.8)	12 (21.1)	2 (3.5)	1.000
Skin rash	1(1.9)	0 (0.0)	0 (0.0)	0 (0.0)	-
Infusion reaction	0 (0.0)	0 (0.0)	0 (0.0)	0 (0.0)	-

—is unable to calculate statistics

## Discussion

The results of this study show that the nimotuzumab group had significantly higher 3-year DMFS compared to the control group. Additionally, the 3-year PFS showed an increasing trend, suggesting that concurrent chemoradiotherapy combined with nimotuzumab after neoadjuvant chemotherapy can effectively reduce distant metastasis in locally advanced NPC, and may be beneficial to PFS and OS over time.

Concurrent chemoradiotherapy following neoadjuvant chemotherapy has emerged as the standard treatment for locally advanced NPC, with the TPF regimen recommended as a grade I neoadjuvant chemotherapy [13]. Although induction chemotherapy combined with concurrent chemoradiotherapy has achieved good results in the treatment of locally advanced NPC, distant metastasis remains a major cause of treatment failure [8]. Hence, further reduction of distant metastasis and improvement of the OS rate are important and difficult issues in the treatment of locally advanced NPC.

EGFR is highly expressed in 80%-90% of patients with NPC [10]. Activation of EGFR can promote proliferation, invasion, and metastasis of tumor cells, as well as inhibit apoptosis of tumor cells, thus inducing tolerance to radiotherapy and chemotherapy [14, 15]. At present, EGFR has become a therapeutic target for NPC. Huang et al. confirmed that the addition of radiotherapy to nimotuzumab improved the overall efficacy of NPC treatment compared to radiotherapy alone [16]. Wu et al. also confirmed that radiotherapy combined with nimotuzumab can improve the efficacy of treatment for locally advanced NPC compared with radiotherapy alone [17]. In a retrospective study conducted by Lu et al., concurrent chemoradiotherapy combined with nimotuzumab was demonstrated to improve DMFS and OS compared to concurrent chemoradiotherapy in patients with NPC and cervical lymph node metastasis [18]. Sun et al. conducted a prospective, randomized, controlled, double-blind, multicenter phase III clinical trial, which demonstrated that compared with concurrent chemoradiotherapy, nimotuzumab combined with concurrent chemoradiotherapy improved the efficacy of treatment for locally advanced NPC, and the 5-year OS rate increased from 64.3% to 76.9% (*P* = 0.042) [19]. These studies collectively support the efficacy of both radiotherapy alone and concurrent chemoradiotherapy plus nimotuzumab in the treatment of locally advanced NPC. Jiang et al. reported that concurrent chemoradiotherapy combined with nimotuzumab after induction chemotherapy improved the objective remission rate and 5-year PFS compared with concurrent chemoradiotherapy alone [20]. Similarly, a retrospective study by Wang et al. confirmed that concurrent chemoradiotherapy combined with nimotuzumab after induction chemotherapy for locally advanced NPC can prolong DMFS for up to five years. For patients with N2-3, the DMFS, and OS of the nimotuzumab group were significantly prolonged [21], which was similar to the results obtained in this study. Our study further confirmed that nimotuzumab combined with concurrent chemoradiotherapy after neoadjuvant chemotherapy improved the efficacy of treatment for locally advanced NPC.Adverse reactions observed in both the nimotuzumab and control groups were comparable and oral mucosal reactions, radiation dermatitis, as well as other reactions did not increase, similar to the results of a previous study [22].

The cumulative dose of cisplatin in concurrent radiochemotherapy is also debated. Lv et al. revealed no significant association between a 200 mg/m^2^ cumulative dose of cisplatin and improved survival, while a 160 mg/m^2^ cumulative dose of cisplatin may be appropriate [23]. Liu et al. revealed a better curative effect with a > 200 mg/m^2^ than ≤ 100 mg/m^2^ cumulative dose of cisplatin, but was comparable with 100–200 mg/m^2^ cumulative dose in concurrent radiochemotherapy [24]. These findings suggested that the higher cumulative dose of cisplatin did not always indicate a better curative effect. The present study adopted a 160 mg/m^2^ cumulative dose of cisplatin. On the contrary, some studies have revealed a better survival benefit with a higher cumulative dose of cisplatin. Jiang et al. revealed higher 3-year PFS and DMFS in > 200 mg/m^2^ cumulative dose of cisplatin than that in ≤ 200 mg/m^2^ cumulative dose in concurrent radiochemotherapy [25].

The subgroup analysis of this study revealed that patients with locally advanced NPC who were < 60 years old and had a neutrophil/lymphocyte ratio ≤ 4 gained a significant survival benefit when treated with neoadjuvant chemotherapy followed by concurrent chemoradiotherapy combined with nimotuzumab. Patients with male,N3 disease and EGFR expression (+ + / +  + +) also showed a favorable trend in terms of survival benefits. Multivariate regression analysis identified the neutrophil/lymphocyte ratio as a risk factor for disease progression and distant metastasis in locally advanced NPC. The neutrophil/lymphocyte ratio is the ratio of absolute counts of neutrophils and lymphocytes in the peripheral blood, which may represent the balance between the pro-tumor inflammatory state and the anti-tumor immune response. Several studies have found that the neutrophil/lymphocyte ratio is an independent factor for tumor prognosis [26–28], confirming the results of this study. Other prognostic factors for NPC [29–33], such as EGFR expression, EB DNA level, LDH, T stage, and N stage, were not detected in this study, which may be related to the retrospective study,relatively small sample size and short follow-up time.

This study provides clinical evidence to support the benefit of concurrent chemoradiotherapy with nimotuzumab after neoadjuvant chemotherapy for locally advanced NPC. However, this study is limited in that it is a retrospective study with a relatively short follow-up duration. Long-term follow-up to observe the 5-year survival data is required. In addition, multicenter, randomized, double-blind, large-sample prospective studies are needed to confirm these findings.

## Conclusion

Based on concurrent chemoradiotherapy after neoadjuvant chemotherapy for locally advanced NPC, nimotuzumab demonstrated a better complete remission rate, as well as significantly improved DMFS and PFS, with a notable increasing trend. The incidence of adverse reactions was comparable. Therefore, concurrent chemoradiotherapy with nimotuzumab after neoadjuvant chemotherapy may be a preferred treatment strategy for locally advanced NPC.

## Data Availability

The datasets used and analysed during the current study are available from the corresponding author on reasonable request.

## Abbreviations

CTV1: Clinical target volume 1
CTV2: Clinical target volume 2
CR: Complete response
DMFS: Distant metastasis-free survival
EGFR: Epithelial growth factor receptor
EB-DNA: Epstein barr virus deoxyribonucleicacid
GTV: Gross tumor volume
HR: Hazard ratio
LRFS: Locoregional relapse-free survival
LDH: Lactate dehydrogenase
NPC: Nasopharyngeal carcinoma
NLR: Neutrophil-to-lymphocyte ratio
ORR: Objective response rate
OS: Overall survival
PFS: Progression-free survival
PR: Partial response
PD: Progressive disease
SD: Stable disease
TPF: Docetaxel,cisplatin,and 5-fluorouracil
